# Surface distortion of Fe dot-decorated TiO_2_ nanotubular templates using time-of-flight grazing incidence small angle scattering

**DOI:** 10.1038/s41598-020-60899-2

**Published:** 2020-03-04

**Authors:** Neelima Paul, Jean-Francóis Moulin, Gaetano Mangiapia, Armin Kriele, Peter Müller-Buschbaum, Matthias Opel, Amitesh Paul

**Affiliations:** 1 Heinz Maier-Leibnitz Zentrum, Technische Universität München, 85748 Garching, Germany; 2 German Engineering Materials Science Centre at Heinz Maier-Leibnitz Zentrum, Helmholtz-Zentrum Geesthacht GmbH, 85748 Garching bei München, Germany; 3 Walther-Meißner-Institut, Bayerische Akademie der Wissenschaften, 85748 Garching, Germany; 4 Technische Universität München, Physik Department E21, Lehrstuhl für Neutronenstreuung, 85748 Garching, Germany; 5 Present Address: MTA Centre for Energy Research, Hungarian Academy of Sciences KFKI Campus, 1121 Budapest, Hungary

**Keywords:** Nanoscale materials, Nanoscale materials

## Abstract

Physical properties of nanoclusters, nanostructures and self-assembled nanodots, which in turn are concomitantly dependent upon the morphological properties, can be modulated for functional purposes. Here, in this article, magnetic nanodots of Fe on semiconductor TiO_2_ nanotubes (TNTs) are investigated with time-of-flight grazing incidence small-angle neutron scattering (TOF-GISANS) as a function of wavelength, chosen from a set of three TNT templates with different correlation lengths. The results are found corroborating with the localized scanning electron microscopy (SEM) images. As we probe the inside and the near-surface region of the Fe-dotted TNTs with respect to their homogeneity, surface distortion and long-range order using TOF-GISANS, gradual aberrations at the top of the near-surface region are identified. Magnetization measurements as a function of temperature and field do not show a typical ferromagnetic behavior but rather a supermagnetic one that is expected from a nonhomogeneous distribution of Fe–dots in the intertubular crevasses.

## Introduction

Growth processes like self-organization are a very elegant way of obtaining a regular spatial arrangement of nanostructures with a controlled size distribution by focusing their nucleation and growth to well defined regions leading to nanopattering. The resulting nanoclusteral morphology with higher statistical relevance has a strong influence on the physical and chemical properties of the material. However, commonly long-range order is still a dream for such self-assembled nanostructures^1,2^.

In the field of spintronics, formation of magnetic metal-oxide interfaces play a crucial role in designing and optimizing the functionality of devices because it has a strong influence on the electronic and magnetic properties of the interacting layers. Electron density redistributions of the involved species can influence the reactivity due to preparation conditions. For example, single metal atoms can diffuse into the magnetic oxide and act as a dopant^3–6^.

In the case of transition-metal oxides, titanium oxides (TiO_2_) are perhaps the most extensively studied material in the form of inorganic semiconductor templates of TiO_2_ nanostructures in the form of rods, tubes or meshes. Such a nanostructured morphology influences the chemical and physical behavior^7,8^. Deposition of metals on such a semiconductor template changes its morphology depending on the chemical interaction at the interface, and determines the diffusivity. In fact, anatase TiO_2_ nanotubes (TNT) arrays are considered as extremely stable anode materials for use in Li-ion batteries because of their ability to not only reversibly intercalate considerable amounts of lithium, but also have a high enough lithium insertion potential to prevent metallic lithium plating and electrolyte decomposition. The nanotubular structure allows easy Li^+^ ion diffusion into the TNTs due to their high specific area, which results in an excellent rate capability and cycling stability of the battery.

Recent investigations on the morphology of nanotube arrays using advances scattering techniques like grazing incidence small-angle scattering (GISAXS) show well defined lateral correlations over a large sample volume^8,9^. Depth dependence of these lateral correlations can be monitored by time-of-flight grazing incidence small-angle neutron scattering (TOF-GISANS), wherein depth specific information is obtained by varying the wavelengths of the incident neutrons, which have high penetration depth^8^.

Decoration or filling of such TNTs with various types of transition metals (such as Fe, V, Mn, Cr) and metal oxides has been reported earlier^10–12^. On the one hand, the purpose of the dopant species is to extend its light absorption spectra and conversion capacities of TiO_2_ to the visible light energy range. On the other hand, collective behavior of magnetic nanostructured array depends on several factors, such as the state of magnetization, mechanism of switching, type of array and of course the fact that they are not simple dipoles. Since the field resulting from dipolar interaction depends on the magnetization state of each nanostructure, it affects the effective field of the neighboring nanostructures in turn. Moreover, remarkable ferromagnetism around room temperature in Fe-doped TiO_2_ suggests that oxygen vacancies and/or defects can be owed to the source of magnetism^13,14^. However, only little or no work has been reported where such self-structured semiconducting oxides were used as templates. The subsequent growth and structure development of magnetic layers on TNTs can be a very interesting combination.

Here, in the present work, using TOF-GISANS at different wavelengths, we show that magnetic dot-like nanostructures of Fe on TNT templates can be grown with graded morphology. We present an evolution of the dot–like structure with increasing probing depth. We also investigate magnetic correlations associated with such a graded Fe-dotted morphology. The selection of an appropriate high quality template is a crucial factor in its pursuit, which depends on detailed structural investigations and analysis, particularly with depth sensitivity.

## Methods

### Scanning electron microscopy

We have used a field-emission scanning electron microscope (SEM) instrument (Hitachi FE-SEM S4800) at Friedrich-Alexander University, Erlangen to characterize the local morphology of TNT arrays. The microscope was operated at 10 kV.

### X-ray diffraction and X-ray reflectivity

X-ray diffraction (XRD) and X-ray reflectivity (XRR) measurements provide information on the crystalline structure, layer thickness and interface roughness of the samples^15^. The measurements were performed on an in-house Empyrean diffractometer from PANalytical. An intense monochromatic quasi-parallel beam was achieved by using a graded parabolic W/Si mirror with an equatorial divergence less than 0.055° inserted at the incident beam path in combination with a 0.18° parallel plate collimator in front of the detector. A Soller collimator with an opening of 0.04 rad at the incident beam was inserted to reduce the axial divergence.

### Grazing incidence small angle scattering

Characterization and understanding the nanoscience and their technological impact has resulted in an immense growth in the fields of nanoscale research. GISAXS and GISANS have emerged as the essential probes for morphological characterization in a non-destructive way, either on a surface, or embedded in a matrix deep below^16–21^.

**Figure 1:**
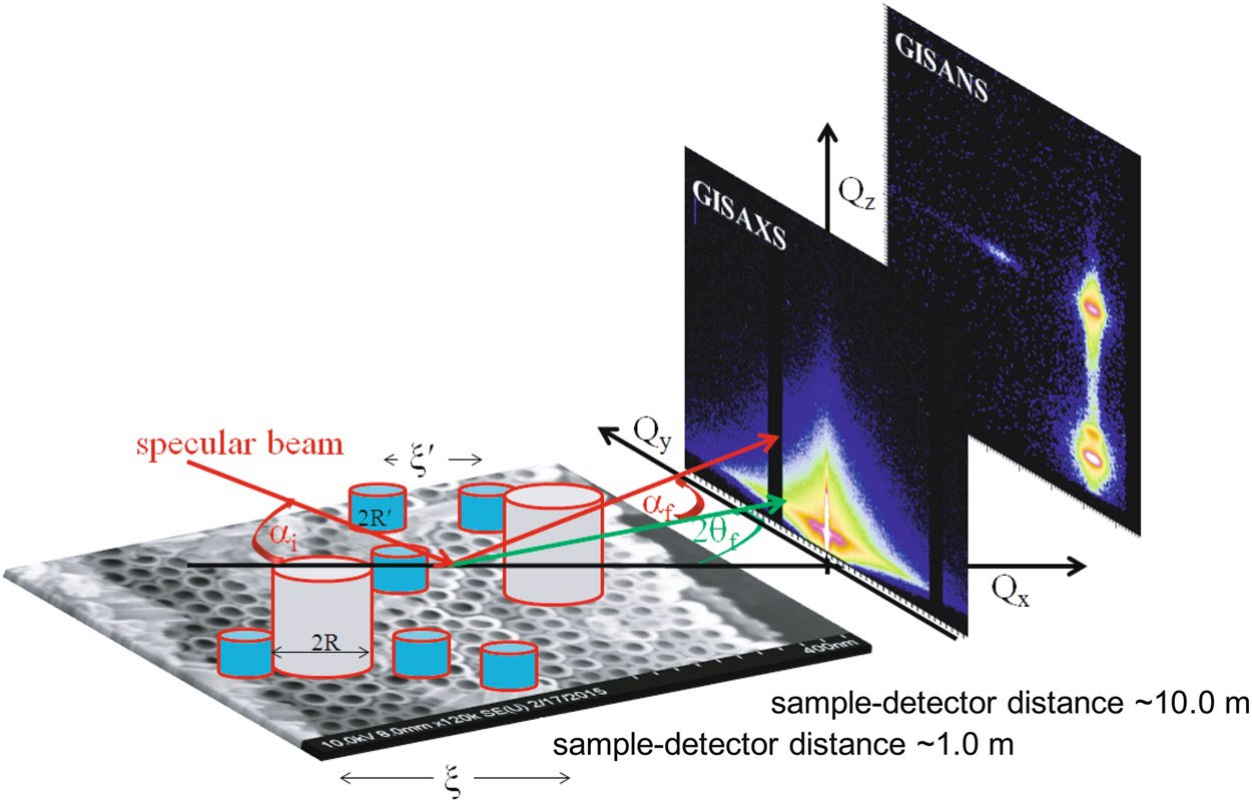
Sketch of the typical scattering geometry for GISANS and GISAXS measurements from a laterally ordered sample. The cylindrical model used for analyzing the GISAS measurements considers scattering objects of sizes *R* and ${R}^{{\rm{{\prime} }}}$ with the distances in between the objects being *ξ* and *ξ*${}^{{\prime} }$, respectively.

GISAXS and GISANS can probe structural film properties perpendicular and parallel to the sample plane with high statistical relevance as compared to microscopic imaging techniques because of the large sample volume probed. Information on film structures perpendicular to the sample plane is usually obtained by the analysis of the specular reflected beam in the *Q*_⊥_ (*Q*_x_, *Q*_z_) direction as explained above. In addition lateral film structures or morphology can be studied by either analyzing scattering parallel to the sample surface at the specular beam position or by analyzing additional off-specular scattering. In Fig. 1 we show the schematic of the scattering geometry.

The total scattering vector is the difference of the wave vector of the incident beam *k*_*i*_ and the scattered beam *k*_*f*_:

1 $$\begin{array}{ccl}\overrightarrow{Q} & = & {\overrightarrow{k}}_{i}-{\overrightarrow{k}}_{f}\\ \left[\begin{array}{c}{Q}_{x}\\ {Q}_{y}\\ {Q}_{z}\end{array}\right] & = & \left[\begin{array}{c}\cos {\alpha }_{{\rm{f}}}\cos 2{\theta }_{{\rm{f}}}-\cos {\alpha }_{{\rm{i}}}\\ \cos {\alpha }_{{\rm{f}}}\sin 2{\theta }_{{\rm{f}}}\\ \sin {\alpha }_{{\rm{i}}}+\sin {\alpha }_{{\rm{f}}}\end{array}\right]\simeq k\left[\begin{array}{c}{\alpha }_{{\rm{i}}}^{2}/2-{\alpha }_{{\rm{f}}}^{2}/2-2{\theta }_{{\rm{f}}}^{2}\\ 2{\theta }_{{\rm{f}}}\\ {\alpha }_{{\rm{i}}}+{\alpha }_{{\rm{f}}}\end{array}\right]\end{array}$$

for low angles, where *α*_i_ is the angle of incidence of the neutrons or X-rays and *α*_f_ is the angle of the scattered wavevector in the *xz* plane (|*k*| = 2*π*/*λ*). In this case *λ* denotes the wavelength and 2*θ*_f_ is the angle in the *xy* plane which is relevant to determine lateral correlation lengths. The angle of incidence chosen was just above the critical angle for total reflection (*α*_*c*_). For specular reflection, $\sin {\alpha }_{i}$ = $\sin {\alpha }_{f}$ = $\sin \alpha$.

Penetration of the beam into the film is of high importance to obtain scattering data, averaging over the structural attributes, perpendicular to the sample plane^22^. When the absorption in the medium cannot be neglected, *Q*_z_ becomes complex and is related to the imaginary part of the refractive index *η* = 1 − *δ* + *iβ*.

2 $${Q}_{{\rm{z}}}=k\{\sqrt{({\sin }^{2}{\alpha }_{{\rm{i}}}-{\sin }^{2}{\alpha }_{{\rm{c}}}+2i\beta )}+\sqrt{({\sin }^{2}{\alpha }_{{\rm{f}}}-{\sin }^{2}{\alpha }_{{\rm{c}}}+2i\beta )}\}$$

Here, *α*_*c*_ is the critical angle and *β* involves the absorption coefficient of the material. For small angles, one can get the values of scattering length density (SLD) from the *α*_*c*_ values following Eq. 3.

3 $${\alpha }_{{\rm{c}}}=\lambda {\left(\frac{SLD}{\pi }\right)}^{1/2}$$

SLD is typically used in the neutron scattering community and is less common in X-ray scattering. The SLD for neutrons is given by SLD = Σ*b*_*i*_/*V*_*m*_ = *ρN*_*A*_Σ*b*_*i*_/Σ*M*_*i*_. Here *b*_*i*_ are the scattering length contributions from the *N* atoms, and *M* is the molecular weight and *V*_*m*_ the volume. *N*_*A*_ is the Avogadro constant and *ρ* is the mass density of the material. This is related to the real part of the complex refractive index *η* = 1 − *δ* + *iβ*, as *δ* = SLD(*λ*^2^/2*π*), where *λ* is the wavelength and *β* = Na_*a*_*λ*∕4*π*, N is the atomic number density, a_*a*_ the absorption cross-section for neutrons as N*b*_*i*_ is the neutron SLD. For X-rays, the SLD is given by a slight variation of the commonly used expression for neutron SLD as the scattering is only due to electrons and they are all identical, therefore the scattering length *b*_*e*_ = (*μ*_0_*e*^2^∕4*πm*)*f*_1_ = *r*_*e*_*f*_1_, where the prefactor *r*_*e*_ = 2.8 × 10^−5^ Å is the classical electron radius, *e* is the charge of an electron, *μ*_0_ is the permeability of free space, *f*_1_ is the real part of the atomic scattering factor and *m* is the mass of an electron. Electron density is *ρ*_*e*_ = (*ρN*_*A*_/*M*)Σ*Z*_*i*_*f*_1_, where *Z*_*i*_ is the atomic number of atom *i*. This is related to the real part of the complex refractive index *η* = 1 − *δ* + *iβ*, as *δ* = (*ρN*_*A*_/*M*)Σ*Z*_*i*_*r*_*e*_(*λ*^2^∕2*π*)*f*_1_ = *ρ*_*e*_*r*_*e*_(*λ*^2^∕2*π*) which is analogous to the expression for neutron SLD. For X-rays, *β* = *μλ*∕4*π*, where *μ* is the linear absorption coefficient.

### Magnetometry

Conventional in-plane magnetization measurements were performed at various temperatures and fields using a superconducting quantum interference device (SQUID) magnetometer from Quantum Design (MPMS-XL).

#### Instruments

TOF-GISANS measurements were performed at the REFSANS instrument of the Heinz Maier-Leibnitz Zentrum in Garching, Germany^23^. Depending on the wavelength, each obtained two-dimensional intensity data set corresponds to a different (*Q*_y_, *Q*_z_) range. Using a single incident angle and a polychromatic incident neutron beam with TOF wavelength resolution, we were able to access selectively several scattering depths/scattering vector ranges. The incident angle at which neutrons impinged on the sample was fixed at *α*_i_ = 0.3°. The sample-to-detector distances were 10.62 m and 5.89 m depending upon the sample templates while the chosen wavelength ranges were 0.32–1.59 nm and 0.29–1.34 nm, respectively. For the Fe-dotted sample, the sample-to-detector distance was 10.33 m while the chosen wavelength range was 0.2–2.2 nm. The beam footprint on the sample was approximately 20 mm in the direction of the beam and 20 mm in the direction perpendicular to the beam. If one neglects the wavelength spread ($\frac{\Delta \lambda }{\lambda }$ ~  5.7%) of neutrons that are incident with a fixed incident angle on the TNT array, then one can roughly estimate that, for wavelengths larger than  ≈0.61 nm, the scattering depth (within the top 100 nm of the film) varies slowly with the wavelength. Thus, a good depth resolution is obtainable for the top 100 nm of the film. For shorter wavelengths, the scattering depth increases dramatically and the full film thickness is probed, without any significant depth resolution.

#### Analysis

For analysis, the raw data of the GISANS spectra have been converted to intensity versus momentum transfer along *Q*_z_ or *Q*_y_ with the software FIT2D version v12.077 for GISANS (DPDAK version v.0.2.9 for GISAXS) using the sample to detector distances of 10612 mm, 5887 mm and 10330 mm for GISANS (1056 mm for GISAXS) and the pixel sizes of the detector. The GISANS and GISAXS data were fitted with the software Genplot version v.2.11 by Computer Graphic Service Ltd.

GISANS/GISAXS data are modelled using an approximation for dense system known as Local Monodisperse Approximation (LMA) which is often used to describe a polydispersed system by separating the form factor from the interference function^1^. The distribution of particles is given by the inter-particle correlation or local interference function S(*q*,*R*). According to paracrystal theory, the scattering function is affected by the shape of aggregation and expressed as a convolution product of S(*q*)*γ(*q*) where *γ*(*q*) is the structure factor of the aggregation by which finite size effects are introduced. S(*q*)*γ(*q*) is obtained by taking the Fourier transform of the products of the correlation functions. Thus the average center-to-center distances (*ξ* and *ξ*${}^{{\prime} }$) of the scattering objects (*R* and *R*${}^{{\prime} }$) were associated with the structure factors and were also obtained from the fits. The distributions of radii (Δ*R*) and standard deviations of correlation lengths (*σ*) were also used as fit parameters^24^.

### Samples

The synthesis of TiO_2_ nanotubular templates was carried out by a low-cost conventional electrochemical anodization. The structures of 1000 nm-thick TNT arrays were formed from metallic Ti films deposited on silicon (SiO_2_) wafers and used as substrates. The details of the sample preparation were discussed earlier by Roy *et al*.^7^. The prepared TNTs were with pore sizes of around 20 nm, 40 nm and 70 nm (inner diameter) having inter-tube distances of about 20–25 nm (bare TNT: 25 nm), 40–50 nm (bare TNT: 50 nm) and 70–80 nm (bare TNT: 80 nm), respectively. The nanostructured templates of bare TNT: 50 nm sample was later used for depositing Fe films. Fe film of 1.0 nm was grown by DC magnetron sputtering on top of the TNT templates (Fe-dotted TNT) followed by a capping layer of Au (1.0 nm) by magnetron sputtering. The capping layer was used to protect it from oxidation. The thicknesses were so chosen that the lateral ordering of the TNTs was maintained. Note that the deposition rates for Fe and Au were kept very low at 0.2 nm/s and 0.8 nm/s, respectively.

## Experimental results and discussion

Earlier, the film morphology of *mesoporous* TiO_2_ network films as templates for the self-assembly of Fe was probed by Ziegler *et al*. with GISAXS^25^. The pore sizes in that investigation were about 50 nm with an inter-pore distance of around 100–150 nm. Interestingly, they found a direct correlation of the growth morphology and structure with magnetic response for three different stages of growth.

In this present article, we first present the morphological aspects of the three different TNT samples using SEM, XRR and GISANS and GISAXS. Secondly, we discuss on the aspect of self-organization of a magnetic metal on a structured semiconducting oxide film at a lower length scale, *viz*., Fe dots decorated on bare TNT: 50 nm array (Fe-dotted TNT).

### SEM

**Figure 2:**
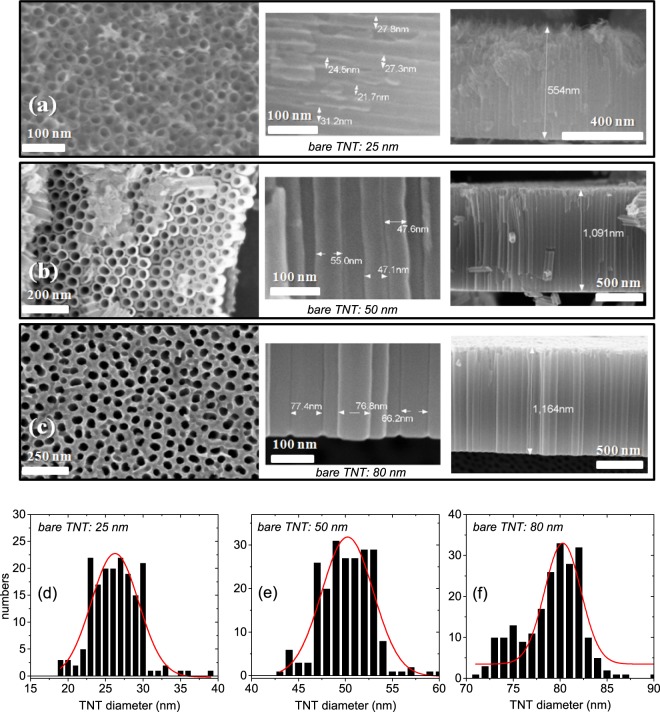
SEM images of the bare TNT arrays. The typical tube sizes are of (**a**) 25 nm (bare TNT: 25 nm), (**b**) 50 nm (bare TNT: 50 nm) and (**c**) 80 nm (bare TNT: 80 nm) for the three samples. The middle panels show the cross-sectional view highlighting the tubular morphology and the dimensions of the bare TNTs. The typical tube lengths for the same are shown in the right panels. (**d**–**f**) Size distribution histograms of the TNTs compiled from the SEM data for bare TNT: 25 nm, bare TNT: 50 nm and bare TNT: 80 nm.

Figure 2(a–c) show the SEM images of the bare TNT arrays with three different tube diameters. The cross-sectional images of the tubes show the corresponding tube diameters of 25 nm (bare TNT: 25 nm), 50 nm (bare TNT: 50 nm) and 80 nm (bare TNT: 80 nm), respectively. The morphologies indicate that the TNTs are rather regular in shape. Additionally, the images show that the typical tube lengths are roughly around 500 nm (for bare TNT: 25 nm) and around 1000 nm (for bare TNT: 50 nm and bare TNT: 80 nm). Some inhomogeneities can be seen for the bare TNT: 25 nm sample, particularly at the top portion of the tubes, as compared to the other two samples. Distortions of the nanotubes at the surface are far less in the bare TNT: 50 nm sample. The most perfect nanotubes from the top to the bottom are visible for the bare TNT: 80 nm sample.

In order to elucidate the average TNT diameters we show the size histograms compiled from the SEM data in Fig. 2(d–f). The statistical analysis of the sets of best-fit models was done using the software PEBBLES^26^ followed by a Gaussian fit to the size distribution in estimating the diameter.

### X-ray reflectivity

**Figure 3:**
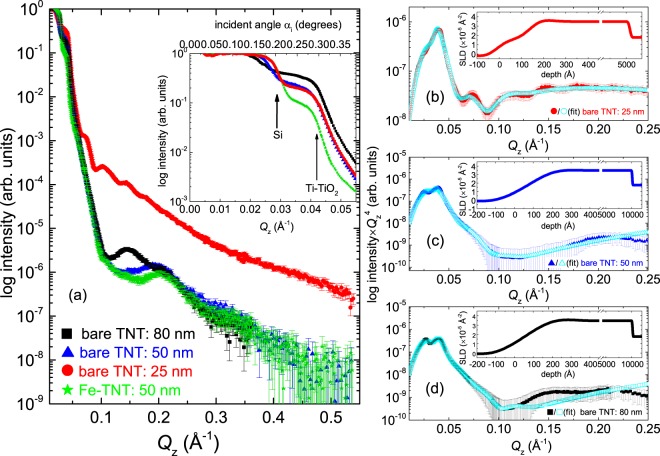
(**a**) XRR patterns from the bare TNT: 25 nm, bare TNT: 50 nm and bare TNT: 80 nm. The seven order intensity drops for bare TNT: 50 nm and bare TNT: 80 nm are noted around *Q*_z_ = 0.1 Å^−1^ (or 1 nm^−1^). The cartoons for the bare TNT template and Fe-dotted TNT are also shown. The zoomed-in inset shows vertical arrows, which indicate the positions of the critical angles *α*_c_s corresponding to the SLDs of porous Si and compact Ti. (**b**–**d**) Reflectivity times *Q*_z_^4^ representation for the TNTs compensating the asymptotic decay of the Fresnel reflectivity. The cyan curves corresponds to the best fit. The insets show SLD profiles obtained from the fits.

The XRR data [*Q*_z_ = *k*(*α*_i_ + *α*_f_)] are shown in Fig. 3(a), which reveal two different critical angles *α*_c_ for each sample (inset of Fig. 3(a)). The theoretical X-ray SLDs of compact Si, compact Ti, and compact anatase TiO_2_ are 2.0 × 10^−5^Å^−2^ (*α*_c_ = 0.222°), 3.55 × 10^−5^Å^−2^ (*α*_c_ = 0.296°), and 3.07 × 10^−5^Å^−2^ (*α*_c_ = 0.275°), respectively. In the present case, the SLDs of the bare TNTs have two distinct values; one is 1.77 × 10^−5^Å^−2^ (*α*_c_ = 0.210°), which represents porous Si and the other is 3.51 × 10^−5^Å^−2^ (*α*_c_ = 0.295°), which lies between the values of anatase TiO_2_ and Ti^8,9^.

One may note that the reflectivity has a drop of seven orders of magnitude within a scattering vector *Q*_z_ range of 0.1 Å^−1^ (or 1 nm^−1^), particularly for bare TNT: 50 nm and bare TNT: 80 nm. Such a rapid decay is associated with corrugated surfaces, typically expected for TNT structures and are not feasible to analyze within the Parratt recursion formulism^15^. The Nevot-Croce correction factor, which is widely used in Parratt’s recursion formula, is valid only in the region *q*_*j*_*σ*_*j*_ <  1 where *q*_*j*_ represents the *z*-component of the wave-vector for a layer *j* with roughness *σ*_*j*_, which restricts *σ*_*j*_ <  10 nm. In case of TNTs, the height of the tubes are around 1 *μ*m, which essentially renders the surface roughness high enough from the fits to the XRR data.

Describing the TNTs as a multilayer system and assuming several top layer thickness, we have fitted the XRR data as shown in Fig. 3(b–d). The fitting was performed by using the optical matrix method within the Parratt’s recursion formula, which takes the instrumental resolution into account. The Nevot-Croce correction factor is taken into account by the use of graded interface, modelled by slices. The SLDs and the interfacial roughnesses of the TNTs are used as fitting parameters. The best fit was determined by the minimization of *χ*^2^ goodness of fit. Figure 3(b–d) is plotted in reflectivity times ${Q}_{{\rm{z}}}^{4}$ representation to compensate the ${Q}_{{\rm{z}}}^{-4}$ asymptotic decay of the Fresnel reflectivity. Inset shows the corresponding SLD profiles. The interface roughness of the near-surface layer above TNT is *σ*_*j*_ ≈  10 nm. Each one of the SLD profiles indicate surface modification for the bulk-like TNT structures below 30 nm. Additional inhomogeneities are seen at the near-surface region above 7 nm for bare TNT: 25 nm, corroborating with the SEM images. However, XRR could only provide information on depth resolved SLD variation and not evolution of the lateral structuring of the TNTs.

The resolution Δ*Q*_z_ along *Q*_z_ is determined by

4 $$\Delta {Q}_{{\rm{z}}}/{Q}_{{\rm{z}}}=\sqrt{{\left(\frac{\Delta \lambda }{\lambda }\right)}^{2}+{(\Delta \alpha )}^{2}cot{\alpha }^{2}}=\sqrt{{\left(\frac{\Delta \lambda }{\lambda }\right)}^{2}+{\left(\frac{\Delta \alpha }{\alpha }\right)}^{2}}$$

for small angles. In order to resolve reflectivity oscillations for a layer thickness *d*, Δ*Q*_z_ must be ≤ 2*π*/*d*. This allows high spatial resolution of a few angström along the depth direction via the uncertainty relation

5 $$\frac{\Delta d}{d}=\frac{\Delta {Q}_{{\rm{z}}}}{{Q}_{{\rm{z}}}}.$$

For a negligible wavelength distribution, the resolution Δ*Q*_z_ and Δ*Q*_y_ are expressed as

6 $$\Delta {Q}_{{\rm{z}}}\approx k(\Delta {\alpha }_{{\rm{i}}}+\Delta {\alpha }_{{\rm{f}}})\approx 1.5\times 1{{0}^{-3}{\rm{\mathring{\rm A} }}}^{-1}\,{\rm{and}}\,$$

7 $$\Delta {Q}_{{\rm{y}}}\approx 2k(\Delta {\theta }_{{\rm{f}}}+{\alpha }_{{\rm{f}}}{\theta }_{{\rm{f}}}\Delta {\alpha }_{{\rm{f}}})\approx 1.5\times 1{{0}^{-3}{\rm{\mathring{\rm A} }}}^{-1}.$$

A difference in the critical angles of Si and anatase TiO_2_ corresponds to a *δQ*_z_ = 1.3$\times 1{{0}^{-2}{\rm{\mathring{\rm A} }}}^{-1}$, and thus can be easily resolved in XRR measurements.

For bare TNT: 25 nm, we find an extended *Q*_z_ range at least up to 0.25 Å^−1^ (or 2.5 nm^−1^), indicating a smoother top layer of  ≈ 31 ± 2 nm comprised of poorer nanostructures. Hereinafter, we will focus on the bare TNT: 50 nm and the bare TNT: 80 nm samples. We will skip the bare TNT: 25 nm sample as the TNTs possess a comparatively higher degree of inhomogeneity towards the top, which is also visible in the SEM micrographs (Fig. 2(a)).

## TOF-GISANS data analysis (bare TNT: 50 nm and bare TNT: 80 nm)

### Two-dimensional data

**Figure 4:**
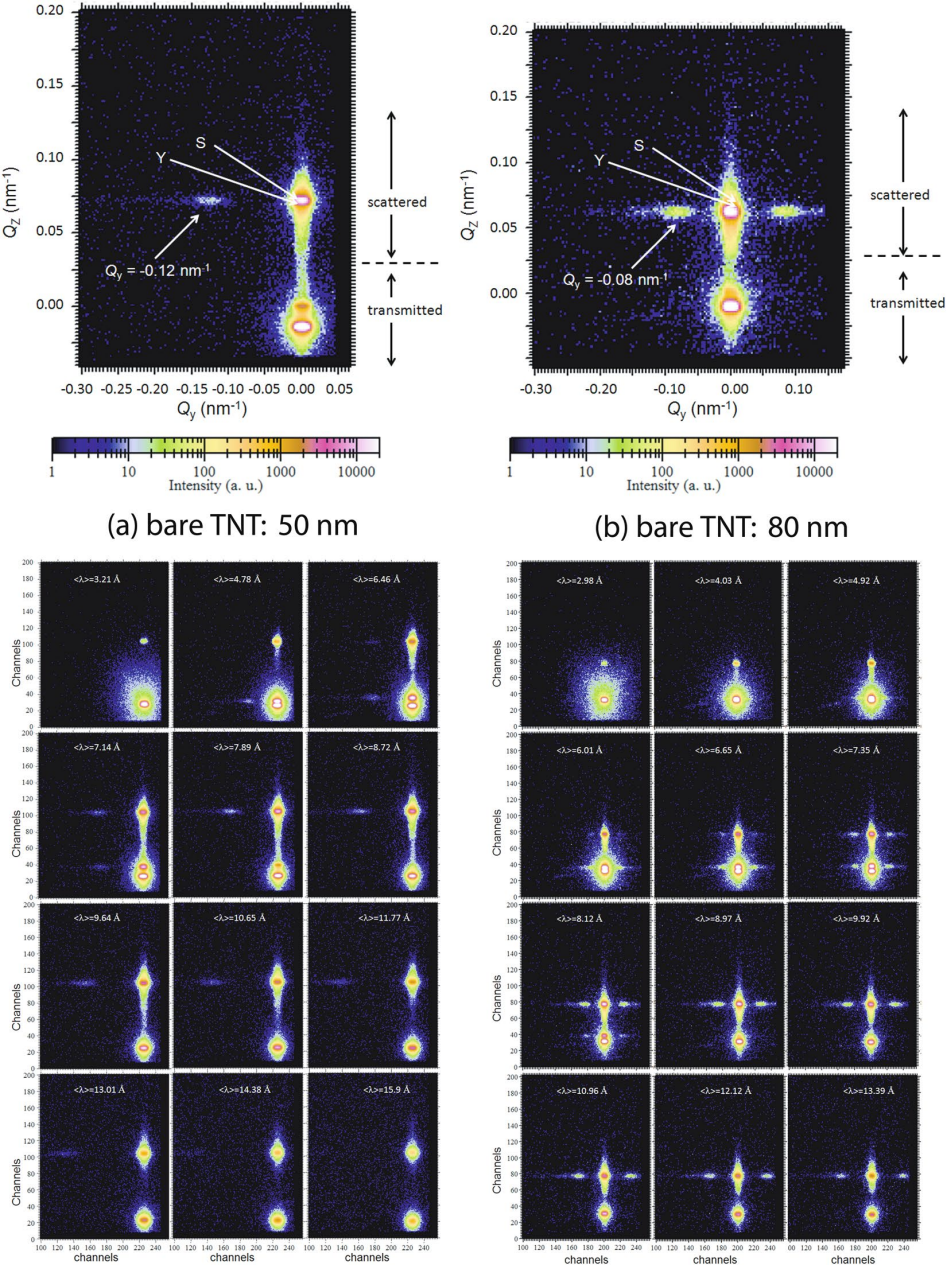
Two-dimensional GISANS data of the bare TNT arrays with the tube diameters of (**a**) 50 nm (bare TNT: 50 nm) and (**b**) 80 nm (bare TNT: 80 nm) are shown for $\left\langle \lambda \right\rangle$ = 0.789 nm and $\left\langle \lambda \right\rangle$ = 0.897 nm. The incident angle was kept at *α*_i_ = 0.3°. The Yoneda (Y), the expected specular (S) and the side peaks are indicated by arrows. We have used a beam stopper to block the direct beam. Shown below are the selected 2-D GISANS images of the TNTs at different wavelengths depicting the evolution of the side peak.

Selected 2-D TOF-GISANS images of the bare TNT arrays measured at $\left\langle \lambda \right\rangle$ = 0.789 nm and $\left\langle \lambda \right\rangle$ = 0.897 nm are depicted in Fig. 4(a,b) for the respective tube diameters of 50 nm (bare TNT: 50 nm) and 80 nm (bare TNT: 80 nm). At these wavelengths, the scattering depth is limited approximately to the top  ≈  15 nm^8^. The scattering signal from the transmitted beam is visible in addition to that from the weak reflected beam. The intense transmitted beam is blocked by a rectangular beamstop to avoid oversaturation of the detector while the reflected beam is allowed.

For both samples, the Yoneda peak (maxima in the Fresnel transmission coefficients (Y)), which is material dependent is clearly seen. However, the specular peak (S), which depends on the incident angle only, remains obscure or absent. This is due to the very weak specular peak from the extremely rough corrugated nanostructures as was evident from the absence of usual X-ray reflectivity profiles from these samples.

The diffuse scattering intensity along the Yoneda peak or the side peak (first order) indicates the presence of a well defined lateral structure which is due to the long-range (nearest-neighbor) order of the bare TNTs with the respective tube diameters. The narrower these peaks are, the better defined is the lateral structure (self-organization). The widths of the profiles along *Q*_z_ at *Q*_y_ = 0.0 nm^−1^, principally give the information on their vertical correlation. The positions of the side peaks at scattering vectors *Q*_y_ = 0.12 nm^−1^ and 0.08 nm^−1^ at *Q*_z_ = 0.07 nm^−1^ for both samples indicate that the sizes of the inter tubular correlation lengths are ≈ 52.33 nm and 78.5 nm, respectively for bare TNT: 50 nm and bare TNT: 80 nm.

Also shown in the lower panels of Fig. 4(a,b) are the selected 2-D TOF-GISANS images of the TNTs at different wavelengths ranging from 0.32–1.59 nm (bare TNT: 50 nm) and 0.29–1.34 nm (bare TNT: 80 nm) depicting the evolution of the side peak in both samples. One may note that longer wavelengths above 0.6 nm exclusively probe the film surface, while information on the morphology is averaged over the complete depth for shorter wavelengths. The side peak or the lateral ordering is absent at shorter wavelengths due to the accumulation of some inhomogeneities along the depth of the TNTs. The side peak becomes more pronounced at longer wavelengths (≥ 0.646 nm for bare TNT: 50 nm and ≥ 0.665 nm for bare TNT: 80 nm), or in other words, when only the near-surface of the TNT array is probed, because the scattering depth is then limited to the upper part (22–27 nm). For even longer wavelengths (≥ 0.964 nm), particularly for bare TNT: 50 nm, the side peak gradually weakens, indicating some inhomogeneities also at the very top (~12 nm) of the near-surface region. For bare TNT: 80 nm, the side peak remains visible up to a wavelength of 1.212 nm and weakens above, which again indicates that the nanostructures are distorted towards the top surface (~11 nm). These two scenarios at the near-surface and the very top surface regarding the overall morphology of the two templates, agree well with the corresponding SEM micrographs (Fig. 3(b,c)).

### One-dimensional data

**Figure 5:**
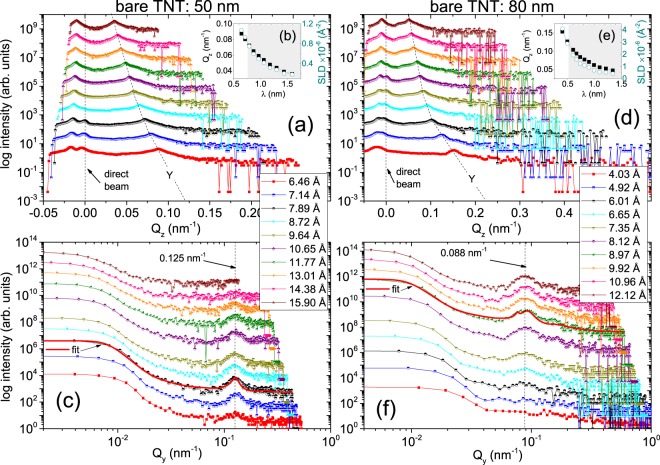
One-dimensional vertical line cuts from the two dimensional GISANS data at different *λ* values along *Q*_z_ of (**a**) the bare TNT: 50 nm and (**d**) the bare TNT: 80 nm samples taken at *Q*_y_ = 0 nm^−1^ values. The direct beam and the Yoneda (Y) peaks are indicated by the arrow and dotted line, respectively. The insets (**b**,**e**) show the evolution of *Q*_z_ and the corresponding SLDs with increasing *λ* values. The shaded regions mark the predominance of surface information. One-dimensional horizontal line cuts from the two-dimensional GISANS data at different *λ* values of (**c**) the bare TNT: 50 nm and (**f**) the bare TNT: 80 nm samples plotted in a log-log presentation. The corresponding fits to the data (solid red lines) are shown for $\left\langle \lambda \right\rangle$ = 0.789 nm and $\left\langle \lambda \right\rangle$ = 0.897 nm which are used in estimating the correlation lengths *ξ* and *ξ*${}^{{\prime} }$ of the cylinders of radii *R* and *R*${}^{{\prime} }$ used in the respective fits. The dashed lines are guides to the eye which correspond to the Y positions in (**a**,**d**) and the side peaks in (**c**,**f**). The curves are shifted vertically along the *y* axis for clarity.

One-dimensional vertical line cuts from the two-dimensional GISANS data at different *λ* values along *Q*_z_ are taken at *Q*_y_ = 0.0 nm^−1^ for the bare TNT: 50 nm and the bare TNT: 80 nm samples and are shown in Fig. 5(a,d), respectively. The positions of the Yoneda peaks (Y) yield the experimentally observed *α*_*c*_ values at different wavelengths (Y = *α*_f_ + *α*_*c*_), where *α*_f_ is the exit angle of the scattered neutrons (see the methods section for details). The line cuts along *Q*_z_ reveal that the Y peak for the two samples are evolving with *λ* and are plotted in Fig. 5(b,e) along with the corresponding SLD values. The theoretical SLD value of compact anatase TiO_2_ is SLD_*theo*(*n*)_ = 2.34 × 10^−6^Å^−2^ (for neutrons). When the material is porous (*e.g*. owing to nanostructuring), depending on the porosity the positions of Y can shift from the theoretical value. Thus, the average porosity of the TNTs is calculated as *P* = 67  ±  4% (60  ±  4%)^8^, estimated from the data at $\left\langle \lambda \right\rangle$ ≈  0.789 nm (0.897 nm) for bare TNT: 50 nm (bare TNT: 80 nm). Note that the scattering information gathered from lower wavelengths of neutrons, carries average information over the entire sample volume with emphasis on the regions buried deep below the surface.

One-dimensional horizontal line cuts from the two-dimensional GISANS data at different *λ* values are the intensity profiles along *Q*_y_ for the bare TNT: 50 nm and the bare TNT: 80 nm samples as shown in Fig. 5(c,f), respectively. The line cuts are taken at the Yoneda peak positions and are plotted in a log-log scale. From the fits to the data we determine the lateral correlation lengths, size distribution and lateral size. The horizontal line cut exhibit a well-established lateral structure (side-maxima) and unresolved large-scale lateral structures (low *Q*_y_ intensity). Representative fits to the data are shown where the side peak is most prominent *viz*., at $\left\langle \lambda \right\rangle$ = 0.789 nm (0.897 nm) for bare TNT: 50 nm (bare TNT: 80 nm). For bare TNT: 50 nm, the value of the radius corresponding to the most prominent cylindrical scattering objects *R* is 24.5  ±  10 nm with a corresponding correlation length *ξ* of 49  ±  3 nm. For bare TNT: 80 nm, the value of *R* is 33  ±  13 nm, which has a *ξ* of 66  ±  5 nm. Additionally, scattering objects at a different lateral length scale are also observed for bare TNT: 80 nm where radius *R*${}^{{\prime} }$ is of 41  ±  10 nm and a correlation length *ξ*${}^{{\prime} }$ is of 82  ±  3 nm. Since *R* and *R*${}^{{\prime} }$ of bare TNT: 80 nm are fairly similar within the error bars, one can infer that the polydispersity index parameter *p* (=$\Delta R/\langle R\rangle$) in bare TNT: 80 nm (*p* = 23/37 = 0.62) is relatively higher than in bare TNT: 50 nm (*p* = 10/24.5 = 0.41). Therefore, even though the TNTs in bare TNT: 80 nm are relatively undistorted along the length and around the surface (SEM), the TNTs in bare TNT: 50 nm are comparatively more monodispersed.

Hereinafter, we will focus on the bare TNT: 50 nm sample. Thus, the Fe dot-decoration has been done upon the bare TNT: 50 nm due the outstanding quality of the TNTs, for example, (i) almost undistorted surface corrugation as evident from the SEM and XRR data, (ii) little or no aberration of the tube lengths as revealed from the depth resolved GISANS information on correlation lengths and (iii) homogeneity or monodispersity as evaluated utilizing the large footprint of neutron beam. All these indicate controlled surface, position and size of TNTs over a large area with a reasonable long-range order.

## TOF-GISANS data analysis (Fe-dotted TNT)

### Two-dimensional data

**Figure 6:**
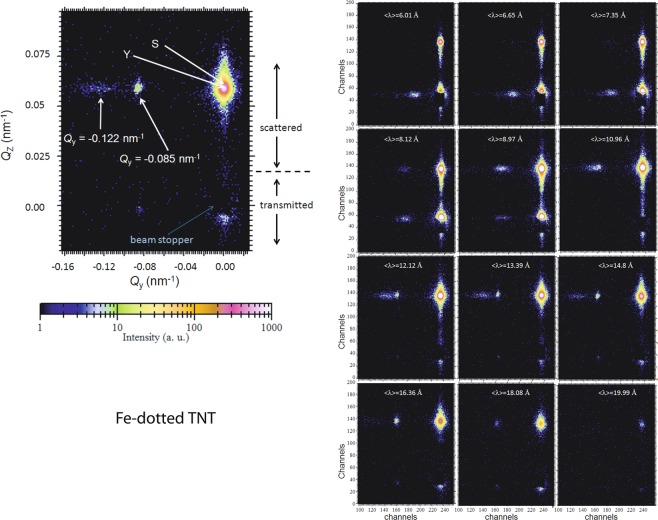
Two-dimensional GISANS data of the Fe-dotted TNT sample is shown for $\left\langle \lambda \right\rangle$ = 1.48 nm. The incident angle was kept at *α*_i_ = 0.3°. The Yoneda (Y), the expected specular (S) and the side peaks are indicated by arrows. We have used a beam stopper to block the direct beam. Shown aside are the selected 2-D GISANS images of the sample at different wavelengths depicting evolution of the side peak.

Selected 2-D TOF-GISANS image of the Fe-dotted TNT arrays measured at $\left\langle \lambda \right\rangle$ = 1.48 nm is depicted in Fig. 6. The diffuse scattering intensity along the Yoneda peak (at *Q*_z_ = 0.07 nm^−1^) or the side peak (first order) indicates the presence of a well defined lateral structure which is due to the long-range (nearest-neighbor) order of the Fe-dotted TNT arrays. The position of the side peak at scattering vectors *Q*_y_ = 0.122 nm^−1^ (primary peak) indicates that the sizes of the inter tubular correlation lengths are  ≈  51.5 nm, which remains fairly unchanged from the Fe TNT: 50 nm sample template. Interestingly, an additional relatively stronger and sharper side peak at *Q*_y_ = 0.085 nm^−1^ (secondary peak) is seen at this wavelength, which corresponds to a inter tubular correlation length of 73.9 nm.

Also shown in the side panels of Fig. 6 are the selected 2-D TOF-GISANS images of the Fe-dotted TNTs at different wavelengths ranging from 0.2–2.2 nm depicting the evolution of the two side peaks. One may note that $\left\langle \lambda \right\rangle$ ≥ 0.61 nm exclusively probe the film surface, while information on the morphology is averaged over the complete depth for shorter wavelengths. The side peaks become pronounced at longer wavelengths (≥ 0.812 nm), or at the surface. For even longer wavelengths (≥ 1.999 nm), the side peaks gradually disappear indicating again inhomogeneous top surface. Between $\left\langle \lambda \right\rangle$ = 1.096–1.636 nm, we see a split of the side peak. This split represents two different long-range orders from the lateral structures.

### One-dimensional data

**Figure 7:**
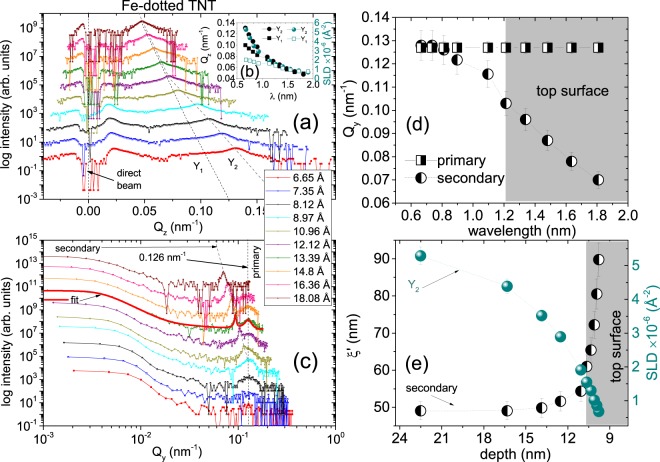
(**a**) One-dimensional vertical line cuts from the two dimensional GISANS data at different *λ* values along *Q*_z_ of Fe–dotted TNT taken at *Q*_y_ = 0 nm^−1^ values. The direct beam and the Yoneda (Y_1_, Y_2_) peaks are indicated by the arrow and dotted lines, respectively. The inset (**b**) shows the evolution of *Q*_z_ and the corresponding SLDs with increasing *λ* values. (**c**) One-dimensional horizontal line cuts from the two-dimensional GISANS data at different *λ* values of Fe–dotted TNT plotted in a log-log presentation. The corresponding fit to the data (solid red lines) is shown for $\left\langle \lambda \right\rangle$ = 1.339 nm which is used in estimating the correlation lengths *ξ* and *ξ*${}^{{\prime} }$ of the cylinders of radii *R* and *R*${}^{{\prime} }$ used in the respective fits. The dashed lines are guides to the eye which correspond to the Y positions in (**a**) and the side peaks in (**c**). The curves are shifted vertically along the *y* axis for clarity. (**d**) The evolution of *Q*_y_ corresponding to the primary and secondary peak positions with increasing *λ* values. (**e**) The plot of correlation lengths *ξ*${}^{{\prime} }$ of the cylinders of radii *R*${}^{{\prime} }$ from the fits to the data corresponding to the secondary peak versus the probing depth with changing *λ*. Also plotted is the evolution of the corresponding SLDs (Y_2_) with increasing probing depth. The shaded regions mark the information from the very top of the near-surface.

One-dimensional vertical line cuts from the two-dimensional GISANS data at different *λ* values along *Q*_z_ are taken at *Q*_y_ = 0.0 nm^−1^ for Fe-dotted TNT and are shown in Fig. 7(a). The positions of the Yoneda peaks (Y) yield the experimentally observed *α*_*c*_ values at different wavelengths (Y = *α*_f_ + *α*_*c*_). The line cuts along *Q*_z_ reveal that there are two Y peaks, Y_1_ and Y_2_, evolving with *λ* and plotted in Fig. 7(b) along with the corresponding SLD values. Y_1_ appears to be rather a shoulder to the stronger Y_2_ peak. The SLD values corresponding to Y_1_ are similar to that of the underlying TNT template. The SLD values corresponding to Y_2_ are similar to that of Au, which represents the top capping layer.

One-dimensional horizontal line cuts from the two-dimensional GISANS data at different *λ* values are the intensity profiles along *Q*_y_ for Fe-dotted TNT as shown in Fig. 7(c). The line cuts are taken at the Yoneda peak positions and are plotted in a log-log scale. For a $\left\langle \lambda \right\rangle$ range between 0.665–0.897 nm, we see the primary peak at *Q*_y_ = 0.122 nm^−1^. A fit to the data gives the value of the radius corresponding to the most prominent cylindrical scattering objects *R*, which is 24.5  ±  16 nm, with a corresponding correlation length *ξ* of 49  ±  3 nm. Thus, the correlation lengths depict the bare TNT: 50 nm template.

For a $\left\langle \lambda \right\rangle$ range between 1.096–1.808 nm, we see the emergence of the secondary side peak, which gradually diverges for larger $\left\langle \lambda \right\rangle$ values. This divergence of the secondary peak shows the changes in structural correlation of the Fe-Au dots as a function of depth. The fit to the data is shown where the two side peaks are seen prominent *viz*., at $\left\langle \lambda \right\rangle$ = 1.339 nm. The value of the radius *R*${}^{{\prime} }$ corresponding to another set of cylindrical scattering objects of 33.5  ±  7 nm, with a corresponding correlation length *ξ*${}^{{\prime} }$ of 67  ±  2 nm. This secondary correlation length represents the Fe-Au dots at the near-surface region.

In order to analyze the growth morphology of the Fe-dotted TNT, we plot the divergent secondary peak positions along *Q*_y_ versus wavelength in Fig. 7(d). The secondary peak merges with the primary peak at $\left\langle \lambda \right\rangle$ = 0.812 nm. In Fig. 7(e), we plot *ξ*${}^{{\prime} }$, which are obtained from the fits to the data as a function of the corresponding accessible probing depth for the near-surface region with changing wavelength. The probing depths ${\mathcal{D}}$ are calculated for the SLD_*theo*_ and *β* of compact TiO_2_ using the equation

8 $${\mathcal{D}}=\frac{\lambda }{\sqrt{2}\pi ({l}_{i}+{l}_{f})}$$

where ${l}_{i,f}={\left[{\sin }^{2}{\alpha }_{{\rm{c}}}-{\sin }^{2}{\alpha }_{i,f}+\sqrt{{\left({\sin }^{2}{\alpha }_{i,f}-{\sin }^{2}{\alpha }_{{\rm{c}}}\right)}^{2}+{(4\beta )}^{2}}\right]}^{\frac{1}{2}}$. Also plotted are the corresponding SLD values from the peak positions along *Q*_z_ evolving with depth. The two curves crosses each other at around 11 nm, beyond which the morphology of the dots changes drastically from the underlying template. This is obviously due to the inhomogeneity at the very top of the near-surface region of the bare TNT: 50 nm template, as has been mentioned in the earlier sections on SEM and TOF-GISANS of the template. This also clarifies the fact that the secondary order is pertained only to the near-surface, whereas the primary order of the rest of the 1 *μ*m TNTs are maintained throughout the depth.

The extent of ${\mathcal{D}}$ and consequently the intensity close to *α*_c_ are smeared by the shape of the angle and the wavelength distributions^27^. Scattered intensity can be a convoluted product of the SLD contrast and the intensity profile of the wave (either evanescent or transmitted) at a specific depth *z*. The intensity of the evanescent wave as a function of wavelength and angle is expressed as

9 $$A(\lambda ,\alpha )={A}_{0}(\lambda ,\alpha )exp(-{\mathcal{D}}/z)$$

where *α* is the angle in the distribution around *α*_i_ and *A*_0_(*λ*, *α*) is the initial intensity of the wave. A negligible angular divergence Δ*α* = 0.0009 is calculated for REFSANS using the equation Δ*α* = arctan (*w*/*l*) where *w* and *l* are the collimation width (made available using two vertical adjustable slits 0–12 mm) and the sample to detector distance (~10 m), respectively. Thus, Δ*λ* becomes the crucial factor for resolving ${\mathcal{D}}$ using neutrons.

**Figure 8:**
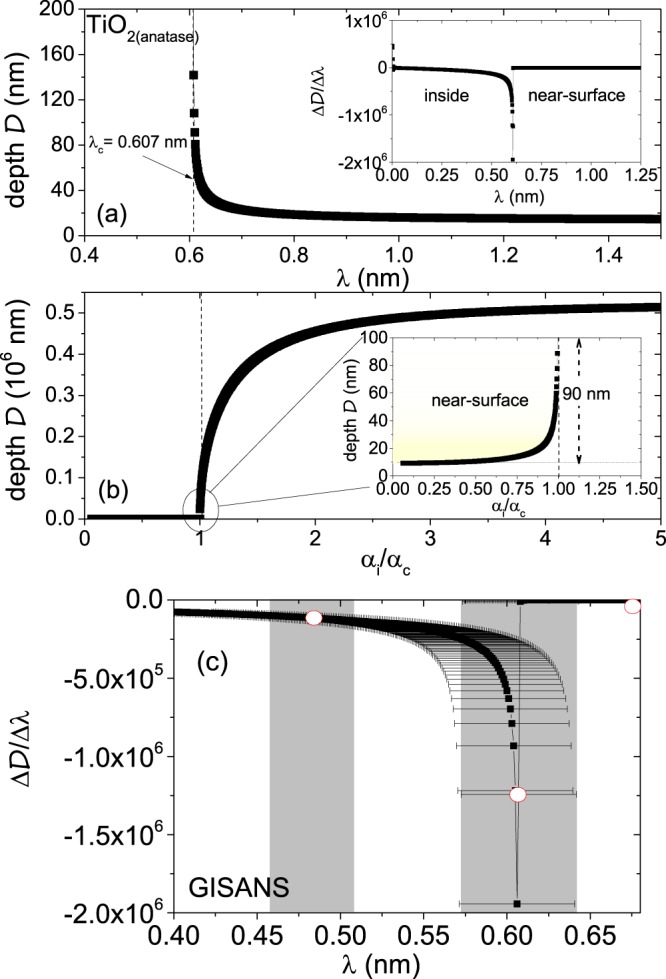
(**a**) Plot of probing depth ${\mathcal{D}}$ versus *λ* for the SLD_*neutron*_ of TiO_2_ (anatase) with atomic number density N = 7.968 × 10^−8^ mol/cm^3^ and absorption cross-section a_*a*_ = 3.5 × 10^−24^ mol/cm^2^. Inset shows the derivative of ${\mathcal{D}}$ versus *λ*. (**b**) Plot of ${\mathcal{D}}$ versus *α*_i_/*α*_c_. Inset shows a zoomed-in scenario of the same around *α*_i_ = *α*_c_, shaded in yellow. (**c**) A zoomed-in scenario of the derivative of ${\mathcal{D}}$ versus *λ* with wavelength spreads in grey. The wavelength spreads are restricted by the wavelength resolution Δ*λ*∕*λ* = 5.7% opted for this TOF-GISANS experiment. The red points mark the wavelengths with non-overlapping wavelength spreads along the profile.

It may be noted that folding the Eq. 8 with a typical wavelength spread (Δ*λ*∕*λ* = 5.7%) function opted at REFSANS, one is sensitive either to the inside or to the near-surface region rather than being sensitive to depth profilometrie. We plot ${\mathcal{D}}$ versus *λ* for TiO_2_ (anatase) and its derivative in Fig. 8(a). The critical wavelength being *λ*_c_ = 0.607  ±  0.035 nm, we predominantly probe the near-surface region for *λ* > *λ*_c_ for the wavelength range explored between 0.2  ±  0.011 nm to 2.2  ±  0.125 nm. One can see that for *λ* = 0.665  ±  0.038 nm (*λ* > *λ*_*c*_), ${\mathcal{D}}$ ≈  22.5  ±  1.3 nm, whereas for *λ* = 0.601  ±  0.034 nm (*λ*_c_ > *λ*), ${\mathcal{D}}$ ≈  71.7  ±  4.1 *μ*m. Thus, a proper depth profiling is not possible with TOF-GISANS at REFSANS in its true sense. The plot of ${\mathcal{D}}$ versus *α*_i_/*α*_c_ and a zoomed-in scenario around *α*_i_ = *α*_c_ are shown in Fig. 8(b). With an increase in *λ* above *λ*_c_ or a decrease in *α*_i_/*α*_c_ ratio, the variation of probing depth becomes more and more slow, which eventually enables us to explore the near-surface region within a limited depth of around 100 nm. A zoomed-in scenario of the derivative of ${\mathcal{D}}$ versus *λ* with wavelength spreads is shown in Fig. 8(c).

### TOF-GISANS and GISAXS

A similar two-dimensional GISAXS data of the bare TNT: 50 nm and the Fe-dotted TNT samples are presented and analyzed in the Supplementary Information. The average porosity estimated from the GISAXS data of bare TNT: 50 nm sample is *P* ≈ 58%. This is similar to the value estimated from the TOF-GISANS data of the same sample, which is *P* ≈ 67%. Both techniques show an average inter-tube spacing (or correlation length) of *ξ* ≈ 50 nm. The size distribution compiled from the SEM data of the sample also show a peak at 50 nm. The polydispersity index parameter *p* are 0.41 and 0.34, as estimated from the TOF-GISANS and SEM data, respectively. X-rays do have a better depth resolution owing to a better monocrhomatization of the wavelength (Eq. 4). However, in a laboratory GISAXS instrument, the tuning is not so precise and we are often restricted with the Yoneda peak position between the sample horizon and the specular peak. This complication can be avoided in TOF-GISANS mode simply by keeping the incident angle fixed and varying the wavelength.

**Figure 9:**
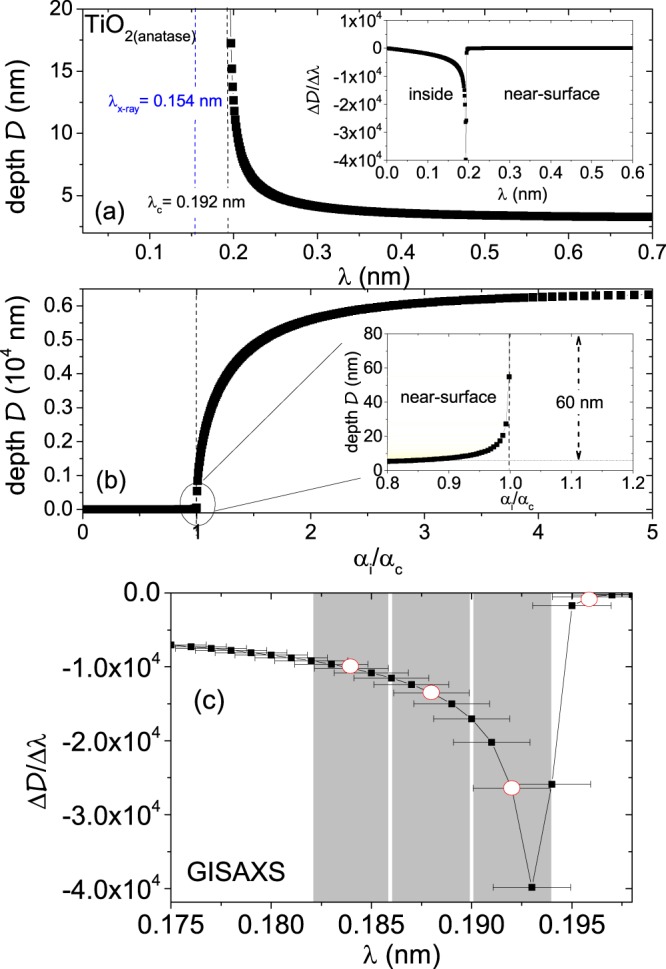
(**a**) Plot of probing depth ${\mathcal{D}}$ versus *λ* for the SLD_*X*−*ray*_ of TiO_2_ (anatase) with linear absorption coefficient *μ* = 7.56 at 8 keV (mass absorption coefficient *μ*_*m*_= 2.023 × 10^2^ cm^2^/g). Inset shows the derivative of ${\mathcal{D}}$ versus *λ*. (**b**) Plot of ${\mathcal{D}}$ versus *α*_i_/*α*_c_. Inset shows a zoomed-in scenario of the same around *α*_i_ = *α*_c_, shaded in yellow. (**c**) A zoomed-in scenario of the derivative of ${\mathcal{D}}$ versus *λ* with wavelength spreads in grey. The wavelength spreads are restricted by the typical wavelength resolution *Δλ*/*λ* = 1% for laboratory GISAXS, the wavelength was however fixed at *λ* = 0.154 nm in our experiment. The red points mark the wavelengths with non-overlapping wavelength spreads along the profile.

For X-rays, the typical wavelength spread (Δ*λ*/*λ* = 1%) function and beam divergence being lower than neutrons, it could have lead to a more sensitive depth profilometrie. The angular divergence Δ*α* = 0.0004 is calculated for GISAXS as well, which again can be considered negligible. Nonetheless, due to a difference in the absorption coefficient (*β*) function, the penetration depth becomes considerably lower for X-rays. We plot ${\mathcal{D}}$ versus *λ* for TiO_2_ (anatase) and its derivative in Fig. 9(a) that can be obtained in principle with a variable wavelength capability. The plot of ${\mathcal{D}}$ versus *α*_i_/*α*_c_ and a zoomed-in scenario around *α*_i_ = *α*_c_ are shown in Fig. 9(b). The critical wavelength being *λ*_c_ = 0.192 ± 0.001 nm, one can probe the near-surface region only for *λ* > *λ*_c_ and the inside region for *λ* < *λ*_c_. A zoomed-in scenario of the derivative of ${\mathcal{D}}$ versus *λ* with wavelength spreads is shown in Fig. 9(c). Our laboratory GISAXS instrument (Ganesha 300 XL) has a fixed *λ* = 0.154 nm. Thus, no depth profiling is possible here. We have therefore shown the GISAXS data in the Supplementary Information, which indeed show comparable results as obtained from TOF-GISANS.

One may note that the SEM data cannot be directly correlated to the GISANS or GISAXS data regarding the information on the object geometry, size distributions and spatial correlations. These differences arise because SEM is a local probe whereas the other two have a depth-resolved or depth-averaged sampling statistics. The penetration depth for GISAXS is calculated at around a few thousand of nanometers for an incident angle *α*_i_ = 0.28° (*λ* = 0.154 nm) on Ti films. For GISANS, the probing depth is not limited, at least for lower wavelengths, lower than *λ*_c_. Thus, both TOF-GISANS and GISAXS could provide corroborative results for our TNTs, except for the top of the near-surface region, which can be depth resolved only by TOF-GISANS. Additionally, the beam footprint in GISANS covers a much larger sample volume as compared to that in GISAXS, which makes its results more realistic.

### Magnetization

#### FC and ZFC

**Figure 10:**
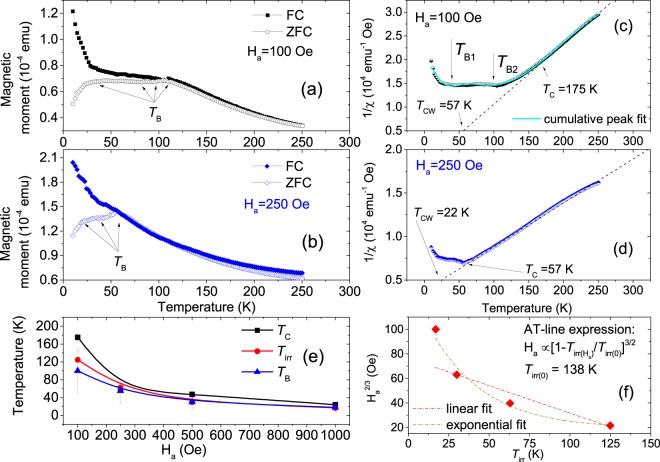
(**a**,**b**) FC-ZFC measurements at 100 Oe and 250 Oe for the Fe–dotted TNT sample at various temperatures. (**c**,**d**) Plot of inverse of differential susceptibilities (1/*χ*(T)) versus temperature and their linear fits following the ZFC DC curves. Gaussian fit to the 1/*χ*(T) curve for ZFC = 100 Oe showing two blocking temperatures *T*_B1_ and *T*_B2_. (**e**) Plot of *T*_C_, *T*_irr_ and *T*_B_ versus field. (**f**) AT-line plot and the corresponding linear and exponential fits.

Transition from ferromagnetism to superparamagnetism (SPM) or superspin-glass (SSG) behavior is generally expected for discrete small clusters (collection of nanoparticles) where the individual magnetic moments within such clusters are thermally unstable. In order to characterize the magnetic properties of the Fe-nanodots indicating supermagnetic-type of behavior, the magnetization (*M*) was measured as a function of temperature (*T*) and are shown in Fig. 10(a,b) for two different applied fields. We used an applied magnetic field **H**_*a*_ = 100 Oe and 250 Oe during measurement before cooling down to 5 K in presence of **H**_*a*_ = 50 kOe (FC). The same protocol was used when cooled down to 5 K in presence of no magnetic field (ZFC). The ZFC curves do not show a typical ferromagnetic behavior but a relatively broad maximum which can be referred to as SPM blocking temperature (*T*_B_) resulting from a nonhomogeneous distribution of either ferromagnetic and/or antiferromagnetic Fe–dots. For ZFC at 100 Oe, we find *T*_B_ ≈  100 K with a temperature distribution *ΔT*_B_ ≈  200 K while for ZFC at 250 Oe, *T*_B_ ≈  55 K with *ΔT*_B_ ≈  40 K.

The DC magnetization data measured at zero Hz frequency is similar to a differential susceptibility (*χ*(T) = d**M**/d**H**) measurement. Therefore, the ZFC data at 100 Oe and 250 Oe can be used to determine the ordering temperatures. We find deviation from linearity at *T*_C_ = 175 K (ZFC at 100 Oe) and 57 K (ZFC at 250 Oe) for the corresponding inverse *χ*(T) plots in Fig. 10(c,d). Accordingly, positive (ferromagnetic) Weiss temperatures *T*_CW_ = 57 K (ZFC at 100 Oe) and *T*_CW_ = 22 K (ZFC at 250 Oe) are obtained from linear fits to the same plots. Furthermore, Gaussian distribution function were used to fit to the *χ*(*T*) curve (ZFC at 100 Oe), which yields two distinct *T*_B_s (*T*_B1_ = 35 ± 20 K and *T*_B2_ = 94 ± 90 K) (Fig. 10(d)), signifying blocking behavior from at least two different particle sizes with their respective distributions.

The *T*_B_ shifts to lower values with increasing field signify lowering of the energy barrier (Fig. 10(e)). An irreversibility temperature (*T*_irr_), where the FC and ZFC curves diverge, could also be identified. The irreversibility temperature corresponds to the SPM transition of the larger particles. A shift of *T*_irr_ to lower temperatures with increasing field is observed to follow the Almeida-Thouless (AT) line that could plausibly indicate SSG behavior (Fig. 10(f))^24^. The zero field SSG freezing temperature *T*_irr(0)_ is estimated at 138 K from a AT-line fit. However, an exponential fit appears to be more suitable in describing the field response, which obviously indicate SPM-type of behavior. Since the number of fields opted for ZFC are limited added with the appearance of multiple *T*_B_s with broad distributions, a conclusive interpretation on the paramagnetic or glassy behavior is rather difficult.

For Fe particles of a fixed size, the magnetization becomes unstable above the *T*_B_, which can be estimated as

10 $${T}_{{\rm{B}}}={K}_{{\rm{u}}}V/25{k}_{{\rm{B}}}$$

where *K*_u_ is the magnetic anisotropy constant (5.5  × 10^5^ erg cm^−3^ ^28^), *k*_B_ = 1.38 × 10^−16^ erg/K is the Boltzmann constant and *V*, the average cluster volume critical for a typical SPM behavior. For *T*_B_ ≈  100 K (ZFC 100 Oe), the particle diameter is 17–20 nm, which is relatively smaller than estimated from the TOF-GISANS data (*R*${}^{{\prime} }$ = 33.5 ± 7 nm). In case where there is a particle-size distribution, the blocking behavior becomes complicated as the relatively smaller particles tend to be blocked, whereas the larger ones still remain unblocked. Fe–dots decoration of intertubular crevasses are responsible for a such particle-size distribution.

#### Hysteresis loop

**Figure 11:**
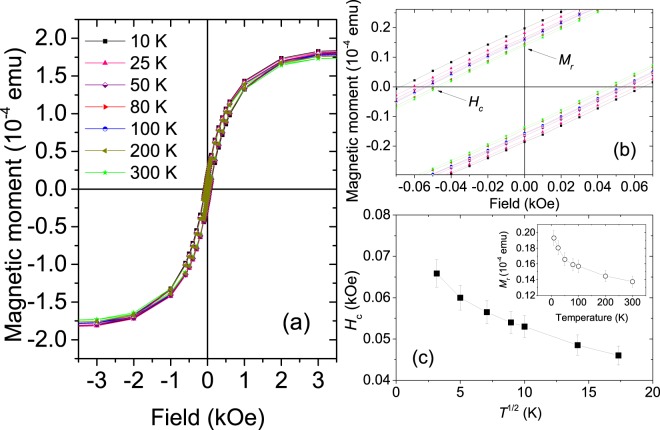
(**a**) Hysteresis loops for the Fe–dotted TNT sample at various temperatures. (**b**) Zoomed-in hysteresis loops for the same showing the temperature evolution of *H*_c_ and *M*_r_. (**c**) Plot of *H*_c_ versus temperature ${T}^{\frac{1}{2}}$. The inset shows a plot of *M*_r_ versus temperature. Error bars represent standard errors of the estimated field and moment values.

In-plane magnetic moment of the Fe-dotted TNT sample was measured as a function of applied filed at seven different temperatures and are shown in Fig. 11(a). We observe a weak ferromagnetic hysteresis. The magnetization is reduced due to an increase in the surface area/volume ratio, causing spin canting at the surface. A zoomed-in version of the same around the coercive fields is shown in Fig. 11(b). The temperature dependence of coercivity (*H*_c_) versus the square root of temperature (*T*^1∕2^) are plotted in Fig. 11(c). Remanent magnetization (*M*_r_) versus temperature also show a gradual decrease (inset of Fig. 11(c)) but does not go to zero even at 300 K. The coercive field can be expressed as

11 $${H}_{{\rm{c}}}=2\frac{{K}_{{\rm{u}}}}{{M}_{{\rm{s}}}}\left[1-{\left(\frac{T}{{T}_{{\rm{B}}}}\right)}^{\frac{1}{2}}\right]$$

for an ensemble of non-interacting particles below their SPM blocking temperature *T*_B_, where *K*_u_ is the anisotropy constant and *M*_s_ the saturation magnetization^29^. However, a nonlinear behavior of *H*_c_ to the *T*^1∕2^ plot and a non-vanishing *M*_r_ for increasing temperatures do not indicate a typical SPM type of behavior^30^. Such a supermagnetic behavior can be due to the filling up of the intertubular crevasses leading to one that is expected for a large particle-size distribution forming clusters of nano–particles. Further elucidation of the magnetic behavior is beyond the scope of the present investigation.

## Summary and Outlook

We report lateral ordering of self-assembled metallic nanodots on inorganic templates such as TNTs with three different structure sizes *viz*., 25 nm (bare TNT: 25 nm), 50 nm (bare TNT: 50 nm) and 80 nm (bare TNT: 80 nm). The bare TNTs are compared with respect to their homogeneity, surface corrugation and long-range order as a function of wavelength using TOF-GISANS for the near-surface region and in depth. Most interestingly, an evolution of the dot–like structure with increasing probing depth is presented using TOF-GISANS. The results are corroborated with those obtained using SEM. The bare TNT: 50 nm sample is chosen for Fe-dot decoration, where it is demonstrated that the lateral ordering at the near-surface has resulted in a graded morphology. Finally, we discussed magnetic correlations as a function of temperature and field. The correlations indicate a nonhomogeneous distribution of Fe–dots in the intertubular crevasses. Instead of a typical ferromagnet their behavior can be termed as supermagnetic-type. Inter-dot correlations of such graded magnetic-dots, when the template itself possesses functional properties like ferroelectricity or photocatalysis, would help us manipulate magnetic properties using non-magnetic means like electric field or photons.

## Supplementary information

Supplementary Information.
