# Supplementary material for: Surface distortion of Fe dot-decorated TiO2 nanotubular templates using time-of-flight grazing incidence small angle scattering
Source: Sci Rep. 2020 Mar 4;10:4038. doi: 10.1038/s41598-020-60899-2 (PMC7055330; doi:10.1038/s41598-020-60899-2)
Supplement: Supplementary file 1 — Supplementary Information. [file 41598_2020_60899_MOESM1_ESM.pdf]

Supplementary Information: Surface distortion of Fe dot-decorated TiO_2 nanotubular templates using time-of-flight grazing incidence small angle scattering [†]

Neelima Paul,[‡] Jean-Francóis Moulin,[¶] Gaetano Mangiapia,[¶] Armin Kriele,[¶] Peter Müller-Buschbaum,[‡] Matthias Opel,[§] and Amitesh Paul[*,∥,⊥]

‡*Heinz Maier-Leibnitz (MLZ), Technische Universität München, Lichtenbergstr. 1, 85748 Garching, Germany*

¶*Helmholtz-Zentrum Geesthacht, Zentrum für Material und Küstenforschung, Außenstelle am MLZ in Garching bei München, Lichtenbergstr. 1, 85748 Garching, Germany*

§*Walther-Meißner-Institut, Bayerische Akademie der Wissenschaften, 85748 Garching, Germany*

∥*Technische Universität München, Physik Department E21, Lehrstuhl für Neutronenstreuung, James-Franck-Straße 1, D-85748 Garching, Germany*

⊥*Present affiliation: MTA Centre for Energy Research, Hungarian Academy of Sciences KFKI Campus 29-33 Konkoly Thege Miklós street 1121 Budapest, Hungary*

E-mail: amitesh.paul@tum.de;amitesh.paul@energia.mta.hu

January 20, 2020

[†] *Corresponding author

Grazing Incidence Small Angle X-ray Scattering

The GISAXS measurements were performed in a noncoplanar scattering geometry to map the lateral correlations using a Ganesha 300 XL SAXS-WAXS system (JJ X-ray systems ApS, Copenhagen/Denmark).[1] The X-ray radiation was produced at 50 kV / 0.6 mA from a Cu anode with a wavelength of $\lambda = 0.154$ nm. The horizontal and vertical beam size was 0.4 mm and 0.3 mm with a divergence of 1 mrad and 0.1 mrad, respectively. The beam footprint on the sample was approximately 15 mm in the direction of the beam and 0.1 mm in the direction perpendicular to the beam. The in-plane angle χ had a range of $\pm 1.15°$. The incident angle was kept at $\alpha_i = 0.28°$. A Pilatus 300 K solid state two–dimensional photon counting detector with a resolution of 172 micron at a detector to sample distance of around 1.056 m was used to record the scattered intensity at room temperature.

For analysis, the raw data of the GISAXS spectrums have been converted to intensity versus momentum transfer Q_z or Q_y with the software DPDAK version v.0.2.9 using the sample to detector distances of 1056 mm and the pixel sizes of the detector. The GISAXS data were fitted with the software Genplot version v.2.11 by Computer Graphic Service Ltd.

GISAXS data analysis (bare TNT: 50 nm and Fe–dotted TNT)

Two-dimensional data: Fig. 1(a,b) show the two-dimensional GISAXS data of the bare TNT: 50 nm and the Fe–dotted TNT sample. The patterns primarily show the specular peak (S) and the Yoneda peak (Y), which are highlighted in the figure. Also shown are the side bands along Q_y at $Q_\mathrm{z} = 0.38$ nm^{-1}.

One-dimensional data: One-dimensional vertical line cuts from the two-dimensional GISAXS data along Q_z are taken at $Q_\mathrm{y} = 0.0$ nm^{-1} for the bare TNT: 50 nm and the Fe–dotted TNT samples and are shown in Fig. 1(c). The line cuts at $Q_\mathrm{y} = 0.0$ nm^{-1} give the vertical correlation lengths of the unresolved structures. The line cuts along Q_z reveal that there are two distinct Yoneda peaks (Y_1 and Y_2) for the two samples. These two Yoneda

peaks are due to the different critical angles (Y = $\alpha_f + \alpha_c$) of the bare TNT: 50 nm sample and the Fe–dotted TNT sample, respectively. The Fe–dotted TNT sample has an additional hump at the position of Y_1, indicating the Yoneda peak of the underlying template. The respective scattering length densities estimated from the values of $Y_1 = 0.325$ nm^{-1} and $Y_2 = 0.352$ nm^{-1} are SLD$_1$ = 1.26×10^{-5}Å^{-2} and SLD$_2$ = 1.85×10^{-5}Å^{-2}, respectively. Thus, the bare TNT: 50 nm sample (Fe–dotted TNT) has an average porosity $P \approx 58 \pm 2\%$ ($\approx 40 \pm 2\%$) when calculated from the SLD of compact anatase TiO_2 (SLD$_{theo(X)}$ = 3.07×10^{-5}Å^{-2} for X-rays).

One-dimensional horizontal line cuts from the two-dimensional GISAXS data are the intensity profiles along Q_y for the bare TNT: 50 nm and the Fe–dotted TNT samples. They are taken at the Yoneda peak positions and are shown in Fig. 1(d) in a log–log scale along with their fits. The most prominent size distribution extracted from the fits to the Q_y line cut data of bare TNT: 50 nm corresponds to an average cylinder radius of $R = 10 \pm 5$ nm and an average inter-tube spacing of $\xi = 50 \pm 10$ nm. The second cylinder has an average inter-tube spacing of $\xi^{'} = 6 \pm 2$ nm with $R^{'} = 3 \pm 1$ nm. These values are in fair agreement with those obtained from the SEM measurements.

After Fe-Au deposition on top of bare TNT: 50 nm (Fe–dotted TNT), the most prominent size distribution extracted from the fits to the Q_y line cut data corresponds to an average cylinder radius $R = 11 \pm 6$ nm and an average inter-tube spacing of $\xi = 55 \pm 14$ nm while $\xi^{'} = 5 \pm 2$ nm with $R^{'} = 2 \pm 1$ nm. The dimensions corresponding to R remain similar to that of bare TNT: 50 nm. Thus the lateral structure of the template remains unaltered.

References

(1) P. Müller-Buschbaum: A basic introduction to grazing incidence small angle X-ray scattering; in Special issue of Lecture Notes in Physics on "Applications of Synchrotron Light to Noncrystalline Diffraction in Materials and Life Sciences", Vol. 776, edt. T.

A. Ezquerra, M. Garcia-Gutierrez, A. Nogales, M. Gomez, p.61-90 Springer Berlin, ISBN-13: 978-3-540-95967-0 (2009).

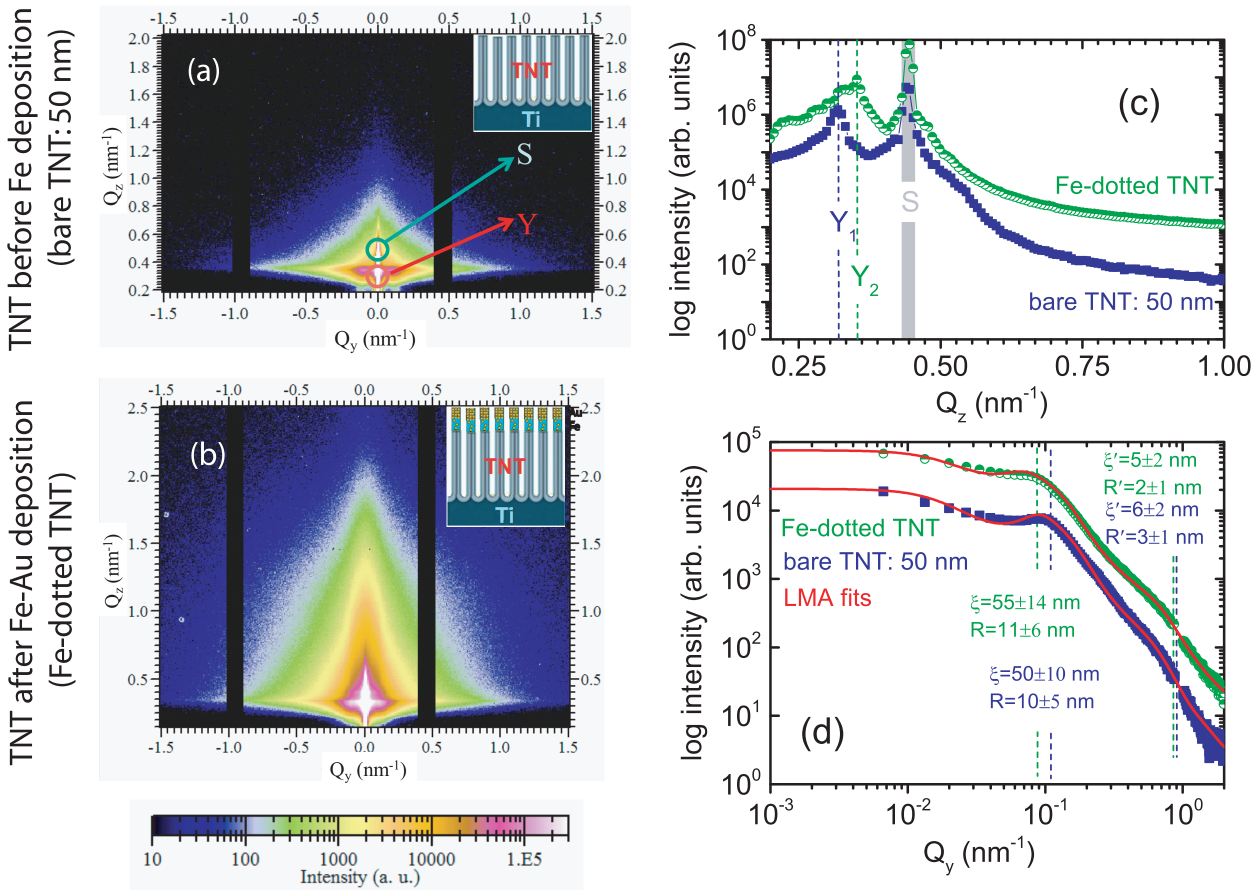

Figure 1 (Color online) Two-dimensional GISAXS data of the TNT arrays (a) before (bare TNT: 50 nm) and (b) after Fe-Au deposition (Fe–dotted TNT) are shown. The incident angle was kept at $\alpha_i = 0.28°$. The vertical black lines are the inter-modular gaps of the Pilatus detector. The specular peak (S) and the Yoneda (Y) peaks are marked with circles and indicated by arrows. Schematic images of the bare TNT: 50 nm and Fe–dotted TNT have been shown alongside. (c) One-dimensional vertical line cuts from the two dimensional GISAXS data along Q_z of the bare TNT: 50 nm and the Fe–dotted TNT samples taken at $Q_y = 0$ nm^{-1} values. The specular peak (S) and the Yoneda (Y_1 and Y_2) peaks are indicated by the shaded region and dotted lines, respectively. (d) One dimensional horizontal line cuts from the two-dimensional GISAXS data of the bare TNT: 50 nm and the Fe–dotted TNT samples plotted in a log-log presentation. The fits to the data (red solid lines) are also shown. The dashed lines correspond to the correlation lengths ξ of the cylinders of radius R used in the respective fits.
